# Longitudinal phenotypic and genomic evidence revealing increased risk of drug resistance accumulation in tuberculosis patients in the counties of central China

**DOI:** 10.1128/spectrum.00380-25

**Published:** 2025-06-23

**Authors:** Fu-Lin Wang, Rong Chen, Qiao Xu, Xiao-Qin Wang, Feng-Xi Tao, Zi-Kang Huang, You-Tong Zhang, Shu-Qiong Chen, Xue-Jing Wu, Hong-Yuan Cao, Qi Jiang

**Affiliations:** 1 Department of Epidemiology and Biostatistics, School of Public Health, Research Center of Public Health, Renmin Hospital of Wuhan University, Wuhan University 572151, Wuhan, Hubei, China; 2 Department of Vaccine, Wuchang District Center for Disease Control and Prevention, Wuhan, Hubei, China; 3 Department of Public Health, Laboratory of Clinical Microbiology, The Second People's Hospital of Xianning City (Xianning Hospital of Tuberculosis Treatment and Control), Xianning, Hubei, China; 4 Department of Public Health, The Fourth Hospital of Wuhan, Wuhan, Hubei, China; ICON plc, London, United Kingdom

**Keywords:** tuberculosis, drug resistance, whole-genome sequencing, transmission cluster, evolution, resistance mutation

## Abstract

Drug-resistant tuberculosis (DR-TB) disproportionately affects rural China, yet the molecular and epidemiological drivers of this disparity remain inadequately explored. This study investigates resistance evolution and transmission dynamics in Xianning, China, using longitudinal data from 3,865 culture-positive pulmonary tuberculosis patients (2016–2023). Phenotypic drug susceptibility testing for 14 commonly used anti-tuberculosis drugs showed a stable multidrug-resistant tuberculosis (MDR-TB) rate of 6.6%, while mono-resistance increased from 8.5% to 12.9% over the study period. Notably, 19.3% (53/275) of patients with ≥2 months of culture positivity developed new phenotypic resistance during treatment. Whole-genome sequencing of strains from the last two years identified resistance accumulation through two additional mechanisms: (i) acquisition of resistance via unfixed mutations in individuals and (ii) transmitted strains harboring novel resistance-conferring mutations absent in parental clones within genomic clusters. For the combined cases of resistance accumulation, multivariable logistic regression revealed that baseline drug resistance increased the risk more than threefold (aOR = 3.65–5.28, varying by resistance type), while rural residence independently doubled the risk (aOR = 2.60, 95% confidence interval:1.11–6.49). Furthermore, three of five genomic clusters with resistance accumulation exhibited urban-rural transmission, highlighting risks linked to cross-district care-seeking. These findings underscore how systemic healthcare barriers in rural China drive DR-TB through both treatment failures and strain transmission. Urgent action is needed to decentralize rapid resistance screening and implement tiered care models in primary clinics to curb transmission and mitigate the expanding DR-TB threat.

**IMPORTANCE:**

The ongoing epidemic of drug-resistant tuberculosis (DR-TB) in resource-poor settings poses a major public health challenge. This study sheds light on the evolution of DR-TB and its community transmission dynamics in central rural China, suggesting that unequal healthcare may exacerbate resistance accumulation risks by driving acquired resistance through inadequate treatment as well as facilitating strain transmission with escalating drug resistance. These findings emphasize the critical need for decentralized, rapid drug-resistance screening, and enhanced diagnosis and treatment strategies in primary care settings, prioritizing vulnerable populations to curb this growing threat.

## INTRODUCTION

Tuberculosis (TB) is the most lethal infectious disease globally and has claimed to more than one billion people over the past two centuries (1). In 2023, the number of cases reached its highest level since the World Health Organization (WHO) began global surveillance in 1995, reaching 8.2 million, with an estimated 1.3 million deaths worldwide (2). Drug resistance is one of the main causes of TB mortality and remains a top priority for high-burden countries, including China. Although only ~5% of the total burden of TB strains are resistant to the main drug rifampicin, the mortality rate is substantially higher and contributes approximately 15%–20% to global TB-related deaths (2). The mechanisms of drug resistance in TB are complex—acquired drug resistance can be established through genomic mutations, and the drug-resistant strains can be further transmitted to secondary cases, leading to community clusters or even epidemic outbreaks (3). There are approximately 15,000 new cases of rifampicin-resistant TB in China each year, with a relatively higher burden in rural areas (2, 4). The epidemiological patterns and causes of the burden of drug resistance remain highly controversial, and there is limited evidence at the county level.

In the clinic, monitoring the drug resistance profile of individual TB patients can detect the accumulation of drug resistance and guide adjustments to treatment regimens. A study conducted in Zhejiang Province, China, tested the serial isolates from multidrug-resistant TB (MDR-TB) patients and found that a quarter of them had increased drug resistance (5). Several cohort studies have also shown the risk of resistance accumulation in patients with other drug resistance (6, 7). However, traditional phenotypic drug susceptibility testing (DST) is time-consuming and labor-intensive, and generally only includes the main first-line drug, rifampicin, and sometimes isoniazid. Comparably, molecular testing, including targeted genetic mutation identification via whole-genome sequencing (WGS), is rapid and can cover a range of therapeutic candidates (8). Inefficient therapy will eradicate susceptible strains but not resistant ones, which will multiply to a fixed frequency, leading to sequential acquisition of resistance (9). The frequency of resistant strains in samples can be detected by WGS, providing molecular evidence for the accumulation of resistance *in vivo*. Moreover, novel mutations indicating resistance accumulation can also be identified in a transmission cluster, such as the genomic analysis in Shanghai MDR-TB patients suggested that two-thirds of clustered patients had evolved with new resistance mutations compared to the parent clone (10).

To systematically analyze the drug resistance accumulation patterns in tuberculosis patients, we used an improved micropore DST method to monitor the full spectrum of resistance to 14 anti-tuberculosis drugs and simultaneously performed WGS of the strains to analyze the molecular processes of resistance accumulation at both the individual and community levels. Combined phenotypic and genomic evidence showed the severity of drug resistance accumulation in tuberculosis patients, especially in patients with initial drug resistance and those living in counties, suggesting the need to strengthen the management of tuberculosis drug resistance in primary care.

## MATERIALS AND METHODS

### Study sites and sample sources

Xianning City is located in the southeastern area of Hubei Province within the central region of China. It encompasses six administrative districts, including a central downtown and five peripheral counties, with a total land area of 9,752 square kilometers and a resident population of 2,612,700 individuals. The prevalence of active pulmonary tuberculosis was reported to be 717.9 per 100,000 residents in the Fifth National Epidemiological Survey in 2010 (11), and the registration incidence decreased from 100 to 60 per 100,000 population in recent years. The overall drug resistance rate was 22.30%, and the MDR-TB rate was 8.15%, higher than the national average (5.7%) (12). This study included the registered active pulmonary tuberculosis patients from 2016 to 2023, whose medical records were derived from the National Registration System of Tuberculosis. Patients were excluded if they were diagnosed with non-tuberculous mycobacterial infections, extrapulmonary tuberculosis, simple tuberculous pleurisy, or had missing data for key variables.

### Laboratory testing methods

All patients diagnosed with tuberculosis at the study site underwent a series of pathogenetic tests, including an antacid smear method, solid culture method, and molecular assay. Positive cultures were sent from the primary laboratories to the center laboratory in the downtown for resistance screening. Patients were followed up monthly during treatment, and positive cultures were also tested for phenotypic resistance. After species identification by the nitrobenzoic acid (PNB) and thiophene-2-carboxylic acid hydrazide (TCH) reactions, *Mycobacterium tuberculosis* strains were then tested by a redox-indicator-based microplate assay (Antu Bioengineering Co., Ltd., Zhengzhou, China) for the susceptibility of 14 anti-TB drugs, including isoniazid (INH), rifampicin (RFP), rifapentine (RFT), streptomycin (SM), ethambutol (EMB), ofloxacin (OFX), levofloxacin (LVF), moxifloxacin (MOF), kanamycin (KAM), capreomycin (CPM), amikacin (AMI), ethionamide (ETO), para-aminosalicylic acid (PAS), and clofazimine (CLO). Of these, OFX, LVF, and MOF are collectively designated as fluoroquinolones (FQs), and KAM, CPM, and AMI are collectively designated as injectable aminoglycosides (AGs). We also used the BACTEC MGIT 960 method to perform PZA susceptibility testing on 197 clinical isolates in 2023. In accordance with WHO definitions (5, 13), we categorized drug-resistant TB into the following groups: Dr-TB (susceptible to INH and RFP but resistant to other drugs), Hr-TB (resistant to INH but susceptible to RFP), multidrug-resistant/rifampicin-resistant TB (MDR/RR-TB), pre-XDR-TB (resistant to RFP and an FQ), and XDR-TB (pre-XDR-TB and resistant to bedaquiline and/or linezolid). As the resistance to bedaquiline and linezolid was not included in phenotypic drug susceptibility testing, making it difficult to differentiate between pre-XDR-TB and XDR-TB, we combined them and referred to these cases collectively as “pre-XDR/XDR-TB” in the later description.

### Whole-genome sequencing and bioinformatic analysis

Genomic DNA was extracted from positive cultures using the cetyltrimethylammonium bromide (CTAB) method (14), and DNA libraries were constructed according to the factory protocol for whole-genome sequencing on the Illumina HiSeq 2500 platform with an average sequencing depth of 100× (15). The sequencing data were analyzed using existing bioinformatics tools and scripts on a Linux system. Sickle (https://github.com/ucdavis-bioinformatics/sickle) was employed to filter low-quality sequencing data, and BWA-MEM (http://bio-bwa.sourceforge.net/) was used to compare raw sequencing data with the reference sequence of the standard H37Rv strain (AL123456.3). Single-nucleotide polymorphisms (SNPs) were identified for each strain using SAMtools. TB-Profiler (16) was employed to identify mutations associated with drug resistance in each strain, and a mutation frequency of greater than 10% was deemed indicative of resistance to the corresponding drug, including INH, RFP, SM, EMB, PZA, LVF, MOF, KAM, CPM, AMI, ETO, PAS, CLO, bedaquiline (BDQ), delamanid (DLM), pretomanid (PA), linezolid (LZD), and cycloserine (CS).

### Genomic cluster identification

After excluding drug-resistant mutations and repetitive sequence regions in the genome, including the PE/PPE family and insertion sequences, MEGA (version XI) was employed to construct a phylogenetic tree for strain samples using the maximum-likelihood method, with a bootstrap value of 1,000 (17). Nodes with a bootstrap value greater than 0.7 were considered accurately structured. Genetic distances between pairwise strains were calculated, and strains with a genetic distance of less than 35 SNPs were considered potential genomic clusters (18). Based on the phylogenetic tree structure, the most recent ancestral node sequence of each cluster was inferred, and the resistance mutations were matched with the strain tip to identify mutations that indicated resistance accumulation (18).

### Reconstruction of dated phylogeny and ancestral trait

Dated phylogeny trees were constructed for transmission clusters by BEAST2 software (v2.7.5), and the geographic locations of ancestral nodes were reconstructed using the beast-classic package (19). Based on the aligned sequences with annotated sampling year and location, 50M MCMC chains were run with the molecular clock of an uncorrelated log-normal distribution at a mean of 1.14 (0.49–1.80) × 10^−7^ substitutions per site per year, the mutation rate for *M. tuberculosis* strains belonging to Lineage 2 (20). The tree priors were set based on the Coalescent Bayesian Skyline model. Priors were adjusted based on the posteriors from rounds of runs until the estimates were convergent. The consensus tree was constructed with the age of the most recent common ancestor (MRCA) node being the height of the tree. Inferred locations of ancestral nodes with probability >0.5 were considered confidential.

### Statistical analysis

Heterogeneous data sets were matched by patient ID, with the analytical data sets anonymized. Data entry and cleaning were performed using Excel 16.0 software, and statistical analysis was performed using R (v4.4.0). The distributions of categorical and continuous variables were compared using the *chi*-square test and the non-parametric rank sum test, respectively. The *chi*-square trend test was employed to ascertain the statistical significance of trends in drug resistance rates over time. The adjusted odds ratio (aOR) and 95% confidence interval (95% CI) for risk factors associated with drug resistance accumulation were calculated using multivariable logistic regression models. A *P*-value less than 0.05 was considered statistically significant.

## RESULTS

### Prevalence of drug-resistant tuberculosis

**Fig 1:**
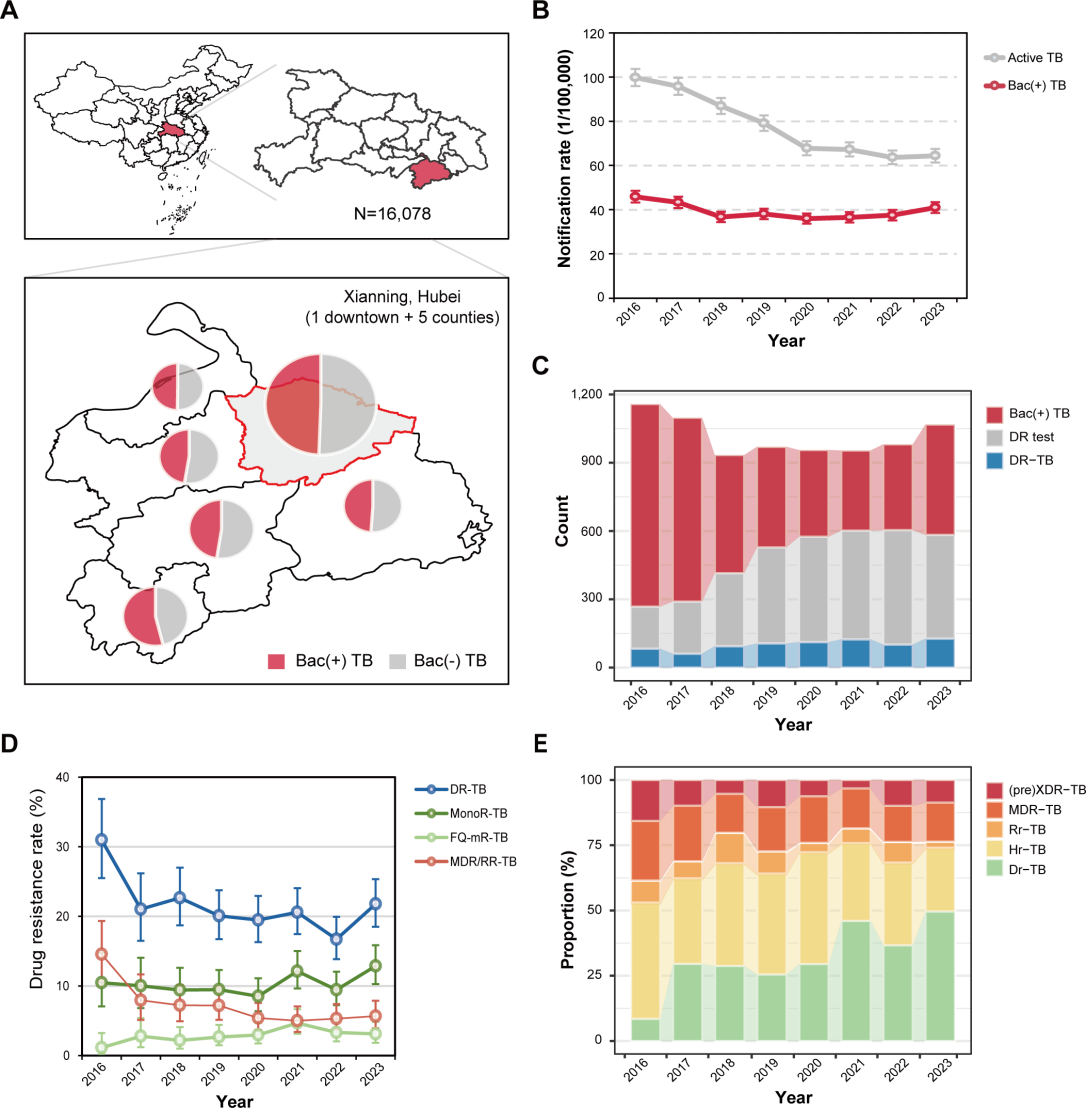
Sample collection and drug resistance prevalence of tuberculosis patients in Xianning, China from 2016 to 2023. (**A**) The geographical distribution of samples and their bacteriological positivity rate. This map was based on the standard map with approval number GS(2020)4621 downloaded from the standard map service website of the Ministry of Natural Resources of China (http://bzdt.ch.mnr.gov.cn/), and the national and regional boundaries have not been modified. (**B**) The annual notification rate of active tuberculosis and bacteriologically positive tuberculosis, marked as Bac(+) TB. (**C**) The number of patients being tested for drug resistance (DR test) and drug-resistant patients (DR-TB), among bacteriologically positive patients. (**D**) The detection rate of different drug resistance types, including DR-TB, mono-resistant TB (monoR-TB), TB with mono-resistance to fluoroquinolones (FQ-mR-TB), and MDR/RR-TB. (**E**) The resistance type composition of drug-resistant patients.

A total of 16,073 patients with active pulmonary TB were registered at the study site, with the numbers in six jurisdictions ranging from 2,041 to 4,272 (Fig. 1A). The median age of all included patients was 53 years (interquartile range: 36–64). The majority of patients were male (70.5%, 11,338/16,073) and farmers (57.7%, 9,269/16,073). Averagely, half of the patients (50.5%, 8,118/16,073) were bacteriologically confirmed, and they were more likely to be the elderly, males, patients with diabetes or TB history, or to have a diagnosis delay than bacteria-negative patients (Table 1).

**TABLE 1:** Basic characteristics of active pulmonary tuberculosis patients in Xianning, 2016–2023

	Total	Bacteria positive	Bacteria negative
	*n* = 16,073	*n* = 8,118	*n* = 7,955
Age (years), n (%)^*a*^			
15–24	636 (3.96)	300 (3.7)	336 (4.22)
25–44	3,757 (23.37)	1,650 (20.33)	2,107 (26.49)
45–64	5,583 (34.74)	2,754 (33.92)	2,829 (35.56)
≥65	6,097 (37.93)	3,414 (42.05)	2,683 (33.73)
Sex, n (%)^*a*^			
Men	11,338 (70.54)	5,840 (71.94)	5,498 (69.11)
Women	4,735 (29.46)	2,278 (28.06)	2,457 (30.89)
Occupation, n (%)			
Farmer	9,269 (57.67)	4,776 (58.83)	4,493 (56.48)
Unemployed	4,361 (27.13)	2,186 (26.93)	2,175 (27.34)
Other	2,443 (15.20)	1,156 (14.24)	1,287 (16.18)
Living area, n (%)			
County	11,804 (73.44)	5,958 (73.39)	5,846 (73.49)
Downtown	4,269 (26.56)	2,160 (26.61)	2,109 (26.52)
Diabetes, n (%)^*a*^			
Yes	530 (3.30)	387 (4.77)	143 (1.80)
No	15,543 (96.70)	7,731 (95.23)	7,812 (98.20)
TB history, n (%)^*a*^			
Retreated	986 (6.13)	899 (11.07)	87 (1.09)
Newly diagnosed	15,087 (93.87)	7,219 (88.93)	7,868 (98.91)
Diagnostic delay, n (%)^*a*^			
>2 months	4,253 (26.46)	2,442 (30.08)	1,811 (22.77)
≤2 months	11,820 (73.53)	5,676 (69.92)	6,144 (76.86)

^*a*^ Statistical significance with the *P*-value in chi-square test <0.05.

During the study period, the registered incidence rate of active TB exhibited a notable decline, while the incidence of bacteria-positive TB demonstrated a relatively stable range, fluctuating between 36.0 and 45.8 per 100,000 residents per year (Fig. 1B). A total of 3,865 patients with positive cultures were tested for phenotypic resistance to 14 anti-tuberculosis drugs, and 20.9% and 6.6% of them were found to be any-drug-resistant and multidrug resistant, respectively. With the drug resistance screening rate increasing, the detection rate of general drug-resistant or MDR-TB appeared to decrease (Fig. 1C and D). However, the detection rate of mono-resistance in bacteria-positive tuberculosis increased in recent years, from 8.5% in 2020 to 12.9% in 2023. The proportion of drug-resistant patients with rifampicin susceptibility has also shown a significant upward trend, as shown in Fig. 1E.

### Molecular characteristics of drug-resistant *M. tuberculosis*

**Fig 2:**
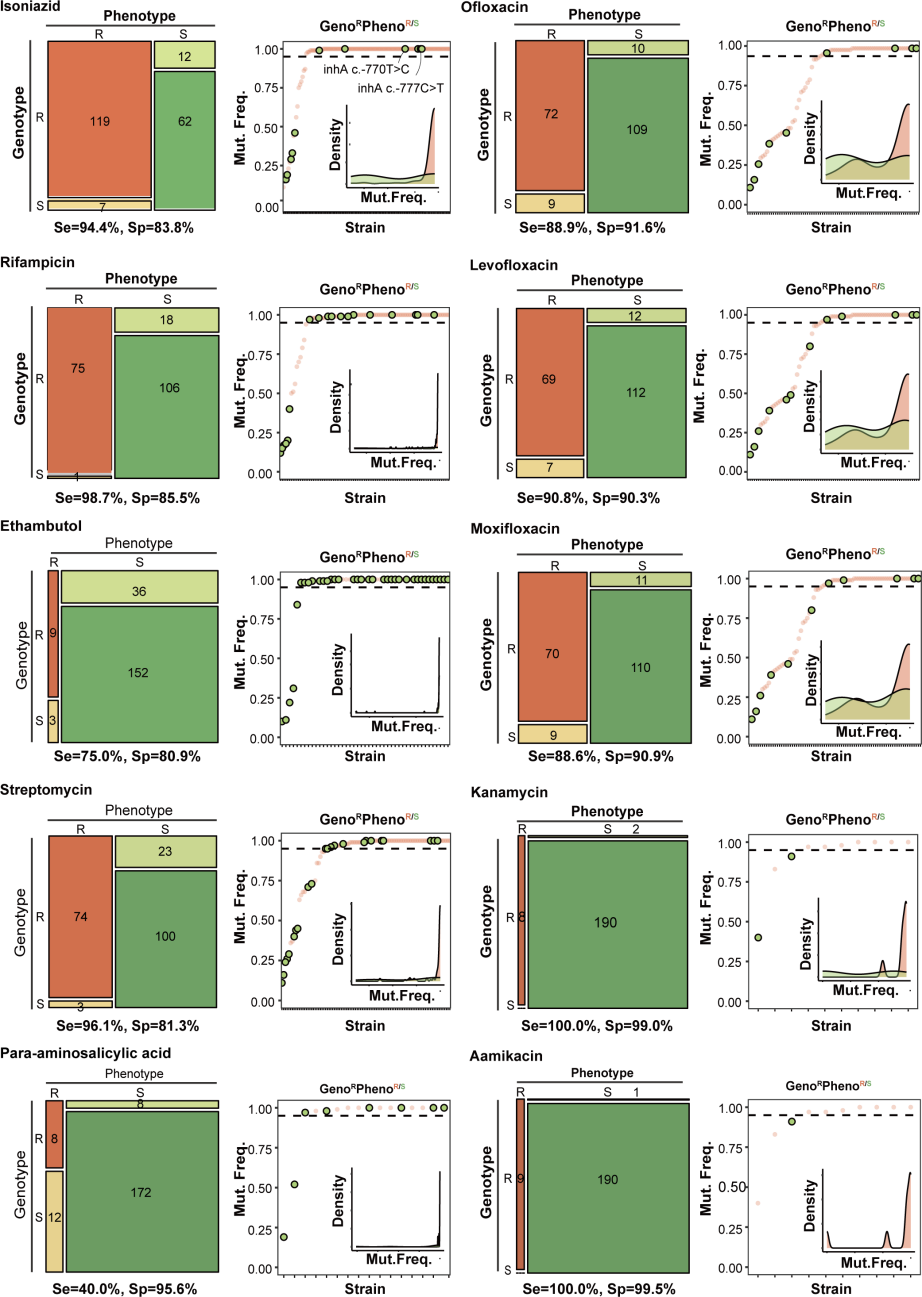
The comparison between drug resistance of phenotypic drug susceptibility testing and whole-genome sequencing. The mosaic plots showed the numbers of phenotypic and genotypic resistance results for each drug, with the sensitivity and specificity labeled below. The right plots for each drug showed the distributions of the frequency of drug resistance mutations in genotypic-resistant samples.

To characterize the molecular features of drug-resistant strains, a total of 201 strains from 2022 to 2023 whose phenotypic resistance to rifampicin, isoniazid, or FQ drugs were subjected to whole-genome sequencing. In comparison to phenotypic resistance, the sensitivities of WGS were 94.4% (95% CI: 88.5–97.5) and 98.7% (95% CI: 91.9–99.9) for predicting resistance to INH and RFP, respectively, and at least 88.6% for second-line drugs except for para-aminosalicylic acid (Fig. 2). Among 197 clinical isolates tested for PZA resistance, 12.7% (25/197) were found to be phenotypically resistant to PZA. To confirm its accuracy, we compared the results to genotypic resistance in 36 isolates that also obtained WGS data. As a result, 20.0% (1/5) and 90.3% (28/31) of the phenotypically resistant and susceptible strains were, respectively, confirmed by WGS genotyping, and another four pheno-susceptible strains were additionally identified as PZA-resistant with mutations in *pncA*. Among 36 pre-XDR-TB/XDR-TB patients with WGS data, only one was further determined to be XDR-TB, resistant to bedaquiline and clofazimine with a G deletion at site 198 of *mmpR5*.

Considering the fact that heteroresistance with low-frequency mutation could be missed by phenotypic resistance testing, we first depicted the frequency of mutations for each strain to delineate the possible reasons for the inconsistent results of drug resistance testing. A considerable proportion of the strains exhibited unfixed mutations with a frequency less than 95%, as shown in Fig. 2. These heteroresistant samples averaged 33.9% (39/115) of the strains with genotypic resistance and phenotypic susceptibility (Geno^R^Pheno^S^) to certain drugs (Fig. 2). Most of these heterogeneous populations had resistant strains with a mutation frequency of less than 50%. In addition, uncommon mutations also contributed to the discordances between genotypic and phenotypic resistance, such as six strains with INH Geno^R^Pheno^S^ carrying mutations in the promoter of the *inhA* gene (c.-770T > C or c.-777C > T), seven strains with EMB Geno^R^Pheno^S^ carrying mutations in the promoter of the *embA* gene (c.-11C > A or c.-16C > T/G), and five strains with SM Geno^R^Pheno^S^ carrying frameshift mutations in the *gid* gene.

### Drug resistance accumulation in individual patients

**Fig 3:**
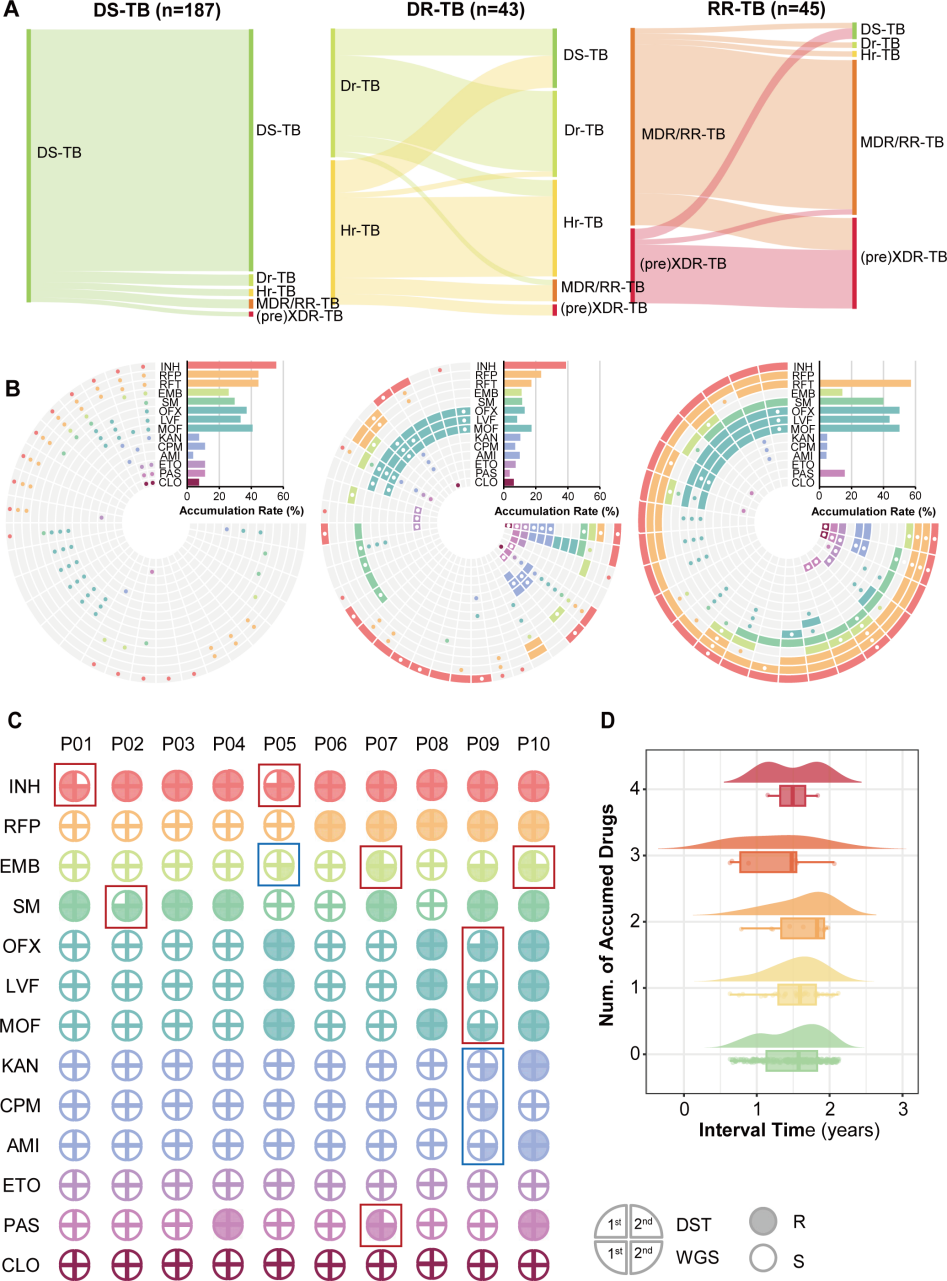
Drug resistance accumulation in individuals. (**A**) Sankey diagram for the changes of resistance profiles during the individual therapy. (**B**) Resistance changes for each drug in patients with resistance accumulation. Gray cells indicate sensitivity to the drug corresponding to each circle, and colored cells indicate resistance. Dots in the cell center indicate the change from sensitivity to resistance or the reverse. The accumulation rate refers to the proportion of patients who develop resistance to a drug among those who were initially sensitive to that drug. (**C**) Phenotypic and genomic resistance of 10 patients with two consecutive isolates. Red boxes marked patients with consistent resistance, except for one phenotypic result of sensitivity (false negative). Blue boxes indicated two patients with emerging resistance identified by WGS: one acquired a mutation conferring resistance to ethambutol (*embB.p*.306 M > I, frequency 31%) and another to aminoglycosides (*rrs.n.1401* A > G, frequency 91%). (**D**) Distribution of the accumulated number of resistance and sampling intervals in paired strains.

Phenotypically, drug resistance accumulation could be captured by monitoring drug resistance at the individual level. In this study, 275 refractory patients were tested as culture-positive at least twice in the therapy course, including initial diagnosis of DS-TB (*n* = 185, 67.3%), Dr-TB (*n* = 21, 7.6%), Hr-TB (*n* = 24, 8.7%), MDR/Rr-TB (*n* = 29, 10.5%), and pre-XDR/XDR-TB (*n* = 16, 5.8%). Accumulation of drug resistance was observed in considerable proportions, with 11.9% (22/185) in initially drug-sensitive patients and 31.1% (14/45) and 37.8% (17/45) among drug-resistant patients with or without resistance to rifampicin (Fig. 3A). Resistance accumulation included both the first-line and second-line drugs in DS-TB patients, while Dr-TB patients mainly accumulated isoniazid resistance, and MDR/RR-TB patients mostly accumulated further resistance to FQ drugs (Fig. 3B).

We also observed 24 drug-resistant patients whose profile changed from resistant TB to susceptible TB, which was confusing in the clinics. To verify the changes, we combined the results of phenotypic and genotypic resistance testing to determine their situation. In ten of the patients with both results for at least two cultures, six patients (with red squares in Fig. 3C), including three cases from phenotypically resistant to sensitive, were identified as consistently resistant TB, regardless of one phenotypic test that presented susceptibility (false negatives). In addition, we captured resistance accumulation in another two patients (with blue squares in Fig. 3C) who depicted novel resistance-conferring mutations in the second isolate. One patient accumulated *embB p*.306M > I (resistance to ethambutol) with a frequency of 31%, and another patient obtained *rrs n.1401*A > G (resistance to aminoglycosides) with a frequency of 91%, which may not necessarily be identified in the routine resistance monitoring.

Overall, these paired strains developed phenotypic resistance to 0–4 drugs during the study period, with no observable correlation between the number of resistance mutations and longer sampling intervals (Fig. 3D). For the 10 patients analyzed, the genetic distances between isolates from the same disease episode ranged from 0 to 17 SNPs, confirming that the genomic variations primarily resulted from resistance accumulation within a single persistent infection.

### Drug resistance accumulation during community transmission

**Fig 4:**
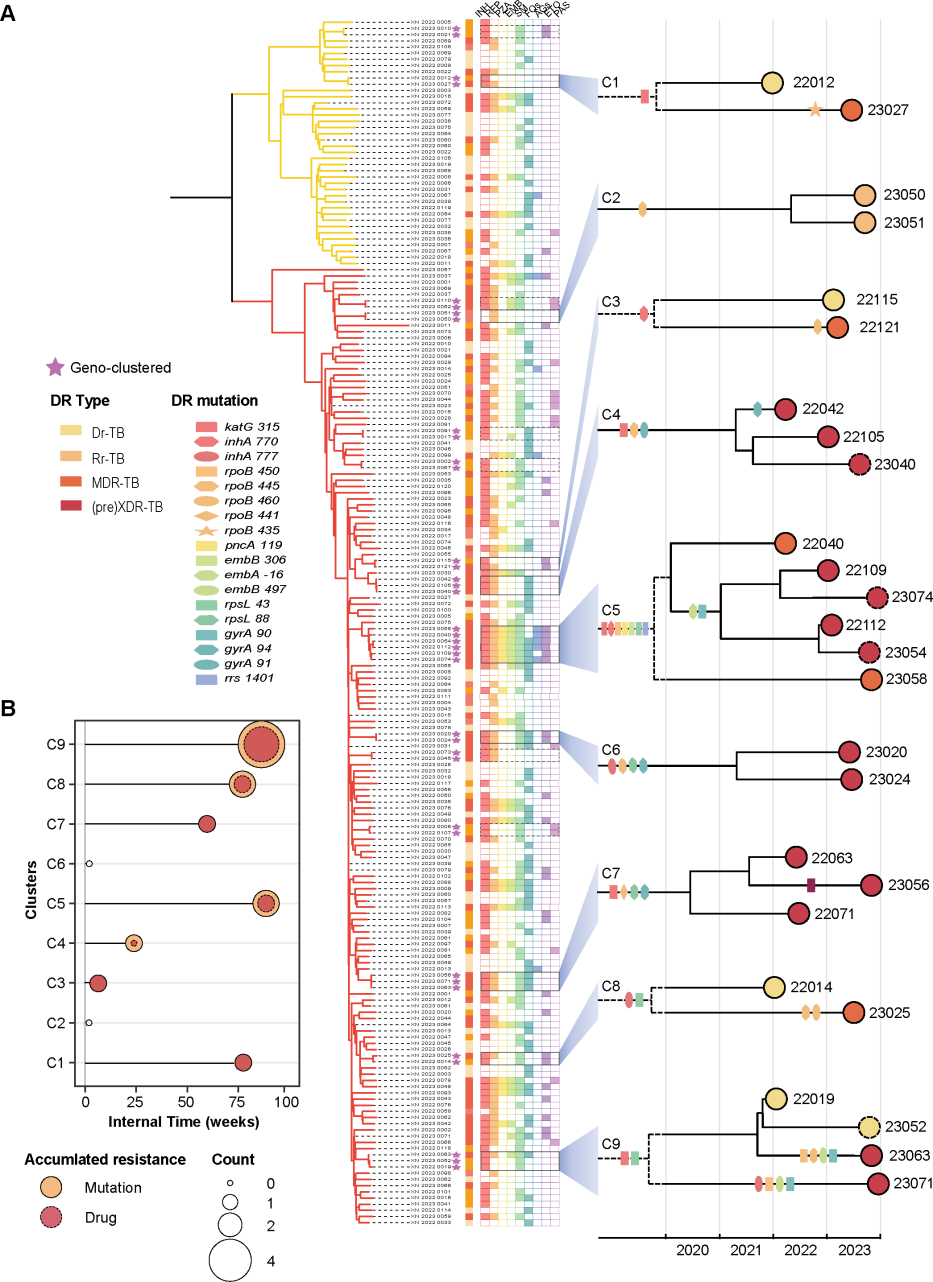
Drug resistance accumulation in transmission clusters. (**A**) Phylogenetic tree of drug-resistant tuberculosis strains with nine transmission clusters expanded in a dated phylogeny. The columns besides the phylogeny represented the genomic clustering, drug resistance type, and resistance to each drug, from left to right: isoniazid (INH), rifampicin (RFP), pyrazinamide (PZA), ethambutol (EMB), streptomycin (SM), fluoroquinolones (FQs), aminoglycosides (AGs), ethionamide (ETO), and para-aminosalicylic acid (PAS). Dashed squares and dots indicated follow-up strains for individuals. (**B**) Distribution of numbers of newly accumulated resistance mutations and resistant drugs relative to time intervals between the source strain and the subsequent strain.

At the community level, resistance accumulation could be inferred if strains carried novel mutations compared to the ancestral sequence. Using the phylogenetic analysis, we identified nine genomic clusters encompassing 26 strains, which indicated the community transmission (Fig. 4A). Among them, five clusters demonstrated resistance accumulation for additional drugs, including four initially Hr-TB clusters and one MDR-TB cluster. The former evolved into MDR-TB (C2, C7, and C9) and pre-XDR-TB (C1), respectively; while the latter one (C5), starting as clones resistant to all four first-line drugs, gained mutations conferring resistance to FQs and AGs and became XDR-TB.

To estimate the evolutionary rate of emerging resistance-conferring mutations, we reconstructed a dated phylogeny for the nine transmission clusters using Bayesian models, with resistance mutations annotated on the branches (Fig. 4A). Except for Cluster 2 and Cluster 6, where strains shared all resistance mutations, strains in the remaining seven clusters acquired 1–4 additional resistance mutations (conferring resistance to 1–3 drugs) over a span of 4–89 weeks (Fig. 4B). Notably, the number of accumulated resistance did not correlate with the time elapsed since divergence from the source strain. This agreed with the findings in the previous analysis of individual phenotypic resistance (Fig. 3D).

**Fig 5:**
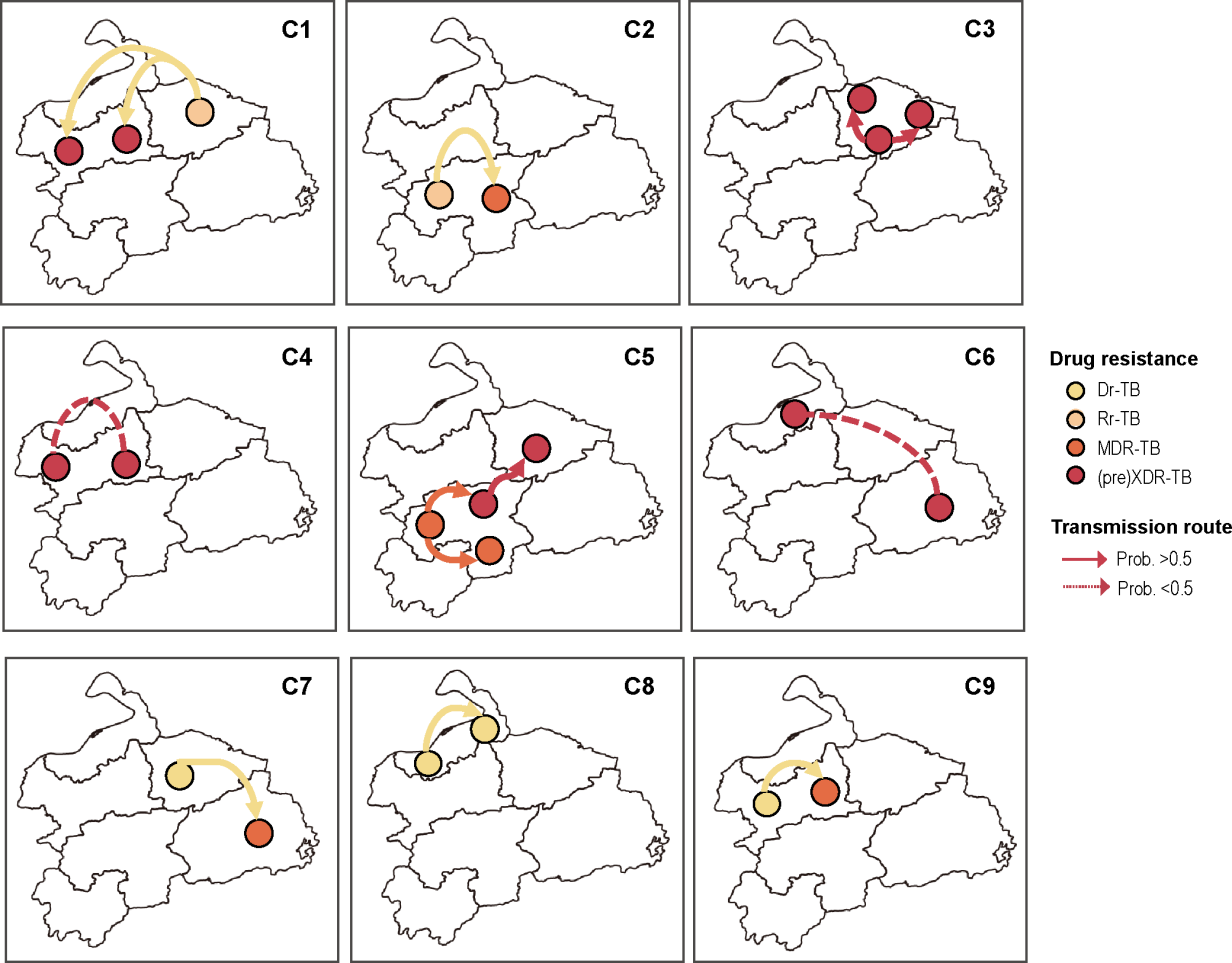
Geographic transmission routes of nine genomic clusters. Solid or dashed arrow lines indicated transmission directions inferred by Bayesian evolutionary models, with the probability of the ancestor node to be >0.5 or <0.5, respectively. This map was based on the standard map with approval number GS(2020)4621 downloaded from the standard map service website of the Ministry of Natural Resources of China (http://bzdt.ch.mnr.gov.cn/), and the national and regional boundaries have not been modified.

To delineate the geographical transmission routes for these clusters, we used Bayesian models to infer the ancestral status of nodes in their time-calibrated phylogenetic trees. Four of the nine clusters spanned multiple regions within the study area, with three clusters (C1, C5, and C7) acquiring additional resistance mutations. Clusters 1 and 7 were inferred to have spread from the urban center to the surrounding counties, whereas Cluster 5 likely originated in a county before reaching the urban center (Fig. 5). Links with probabilities < 0.5 suggested indirect transmission between the genome-clustered patients, possibly due to undetected intermediate cases.

### Risk factors for drug resistance accumulation

To explore the risk factors for resistance accumulation, we determined the cases by both phenotypic evidence (phenotypic resistance changing from sensitivity to resistance) and genotypic evidence (emergence of unfixed mutations conferring drug resistance or novel mutations occurring in transmission clusters) (Table 2). Among the culture-positive patients, accumulation of phenotypical resistance was more likely to occur in retreated patients than newly diagnosed patients, with the adjusted OR of 6.28 (95% CI: 3.18, 12.20), and Hr-TB possessed 3.87 (95% CI: 1.56, 8.74) times the risk than that of pan-susceptible patients, respectively. Interestingly, the diagnosis year was identified as a protective factor, suggesting the improvement of tuberculosis care at the site. Combined with the genomic evidence for resistance accumulation, the risks of retreated patients and drug-resistant patients remained significant among refractory patients, and all the drug resistance types presented high risks of resistance accumulation. Moreover, patients living in the counties had 2.60 (1.11, 6.49) times the risk of downtown patients, which was thought to be related to the cross-district care-seeking in remote areas, as suggested in the transmission pattern above. Given that males were reported to have a higher risk of TB and could be a potential confounder, we also conducted a subgroup analysis stratified by gender to validate the risk factors for drug resistance accumulation. The results showed similar trends, though with reduced statistical significance due to the limited sample size (data not shown).

**TABLE 2:** Multivariable logistic regression models of drug resistance accumulation in tuberculosis patients of Xianning, China^*b*^

	Model 1 (culture-positive patients, *n* = 3,729)	Model 2 (refractory patients, *n* = 275)	Model 3 (drug-resistant patients, *n* = 196)
Age: ≥65 yr (vs. <65)	1.51 (0.78, 2.84)	1.31 (0.64, 2.71)	1.30 (0.63, 2.65)
Sex: men (vs. Women)	2.07 (0.95, 5.20)	1.58 (0.66, 4.09)	1.08 (0.46, 2.61)
Area: county (vs. downtown)	1.15 (0.56, 2.42)	1.36 (0.61, 3.12)	**2.60 (1.11, 6.49**)
Occupation: farmer (vs. other)	1.21 (0.66, 2.25)	0.87 (0.43, 1.76)	0.80 (0.38, 1.65)
History: retreated (vs. new cases)	**6.28 (3.18, 12.20**)	**3.74 (1.67, 8.48**)	**8.23 (2.54, 29.36**)
Diagnostic delay (vs. no delay)	2.57 (0.80, 8.32)	1.33 (0.40, 4.29)	0.28 (0.03, 11.94)
Diabetes (vs. no diabetes)	1.04 (0.16, 3.76)	0.90 (0.16, 3.73)	0.85 (0.26, 2.65)
Diagnosis year (per year)	**0.81 (0.69, 0.94**)	0.96 (0.81, 1.15)	1.04 (0.74, 1.47)
Initial resistance (vs. DS-TB)			
Dr-TB	2.45 (0.70, 6.62)	**5.28 (1.59, 16.54**)	Ref.^*a*^
Hr-TB	**3.87 (1.56, 8.74**)	**4.41 (1.46, 13.16**)	**0.21 (0.09, 0.48**)
MDR/RR-TB	2.62 (0.84, 7.48)	**3.65 (1.33, 9.88**)	**0.19 (0.07, 0.48**)

^*a*^ Dr-TB was set as the control group in Model 3.

^*b*^ Drug resistance accumulation was determined by only phenotypic evidence in Model 1 and by both phenotypic and genomic evidence in Models 2 and 3. Model 3 was performed among drug-resistant patients with whole-genome sequenced strains in the recent 2 years. Statistical significance with *P*-value <0.05 was marked in bold.

## DISCUSSION

This genomic epidemiological study provides a comprehensive analysis of the epidemic burden of pulmonary tuberculosis over 8 years of drug resistance surveillance in a prefectural-level city in central China. The findings reveal a concerning upward trend in general drug resistance beyond rifampicin resistance, with a significant accumulation of drug resistance observed in both initially drug-susceptible and drug-resistant patients. Integrating phenotypic and genomic evidence, the study identifies retreated patients, initially drug-resistant patients, and those residing in counties as high-risk groups for acquiring additional drug resistance and developing more complex, difficult-to-treat forms of TB. Genomic analysis further highlights the spatial dynamics of TB transmission, revealing frequent transmission clusters between the downtown center and surrounding counties. This pattern is exacerbated by the necessity for drug-resistant patients to travel long distances across districts to access centralized medical treatment, potentially facilitating the spread of resistant strains. These findings underscore the urgent need for targeted interventions, including decentralized healthcare services and enhanced surveillance, to curb the growing burden of drug-resistant TB and mitigate its public health impact.

The centralized care for drug-resistant tuberculosis was thought to limit the treatment effects for patients in remote areas and meanwhile increasing the risk of transmission during their frequent cross-regional movements. To improve access to medical services and treatment outcomes, WHO approved a decentralized management approach for drug-resistant tuberculosis in 2011 (21). Successful implementation was reported in South Africa, where decentralized care with qualified services had a shorter time to treatment initiation and better treatment outcomes (22). A meta-analysis estimated an increased odds ratio of 13% for the treatment success of decentralized versus centralized care of MDR-TB (23). Although promoting the decentralization of drug-resistant tuberculosis care faces many challenges, the newest policy for tuberculosis control during 2024–2030 in China advocates for moving drug resistance diagnosis forward to the county level, for at least the core first-line drugs (24). In addition, cooperation among multiple social sectors to improve the accessibility of tuberculosis treatment, including establishing a drug resistance diagnosis platform, ensuring the supply of second-line drugs, sharing expert services, and enhancing policy support, will be conducive to initiating decentralized management and ultimately achieving successful treatment (25).

Overall, we found that 11.9% and 34.4% of patients with initially drug-susceptible and drug-resistant TB, respectively, accumulated phenotypic resistance. Compared with pan-susceptible patients, the risk of resistance accumulation was highest in general drug-resistant patients who were susceptible to isoniazid or rifampicin. Previous studies always focused on MDR/RR-TB patients, among whom the risk of resistance accumulation increased as their initial resistance became more severe (26). This is probably due to less efficient drugs in the second-line routine therapy for those severe patients. While among general drug-resistant patients, the risk mainly comes from the delayed diagnosis or underdiagnosis of the resistance profile. The increasing prevalence of general drug-resistant tuberculosis in the study site also indicated the neglect of these patients by the disease control system. Thus, for both rifampicin-sensitive and -resistant patients, timely and comprehensive resistance testing is especially necessary before anti-TB treatment.

In consecutive samples, we found that 23.5% of DR-TB patients exhibited the phenotype reversion from resistant to sensitive, which has also been observed in other studies (27, 28). This phenomenon is often explained by heteroresistance—a phenotype where bacterial populations contain subpopulations with lower susceptibility to antibiotics compared to the dominant population (29). In patients with heteroresistance, both drug-resistant and -susceptible strains exist *in vivo*. Some strains carrying low-frequency mutations in the bacterial population may go undetected, while some drug-resistant strains may reside in lesions not covered by the sampling process (30), which may contribute to phenotype sensitivity. In our study, among the 10 patients with WGS data of paired strains, resistance-conferring mutations were consistently present, with low-frequency mutations missed by phenotypic DST, supporting the heteroresistance theory. This highlights the importance of continuous resistance monitoring in clinical settings.

Apart from the 10 patients with WGS data of paired strains, we were unable to confirm the consistency for isolates before 2022. This is a common challenge in the clinical setting, and this result has limited significance for clinical drug use, which is not dependent on the strain identity. Therefore, we focus on the evolution of clinical drug resistance to guide therapy, with the aim of using the observed changes in resistance patterns to effectively inform treatment decisions.

The accumulation of drug resistance was also identified in community transmission clusters if the strains harbored novel resistance-conferring mutations compared to the ancestral sequences. In Shanghai, 87% of the MDR-TB clusters accumulated additional drug resistance mutations, which mainly confer resistance to FQ drugs (10). A larger study enrolling a global data set of over 10,000 *M*. *tuberculosis* strains revealed the consistent order of resistance acquisition across countries, based on the time-calibrated evolutionary model (31). Similar to our results, the proportion of acquired drug resistance among MDR-TB varied from 60% to 100% in different countries (31–33).

MDR/RR-TB patients mainly developed further resistance to FQ drugs, which also occurred in refractory rifampicin-sensitive patients. Proportions of FQ resistance among MDR/RR-TB were 40.6% in Xianning, which is similar to the rate in Shanghai (34.7%) (34), Sichuan (33.5%) (35), but significantly higher than that in Zhejiang (22.7%) (36). The proportion among rifampicin-sensitive patients reached 3.2% in our data set, with the mono-resistance rate increasing per year. Limited areas in China have implemented FQ resistance screening among rifampicin-sensitive patients. A 2-year study in Zhejiang Province used WGS to investigate FQ resistance and found that 4.0% of rifampicin-susceptible TB were resistant to an FQ drug (36). Being second-line drugs, FQs are not recommended to treat drug-susceptible TB patients; however, our previous investigation suggested that physicians could prescribe the drug to reduce inflammation before a clear diagnosis, especially in primary care with a lax regulatory environment for antibiotics (37). FQ resistance has become a serious problem in TB control, while the screening rate is relatively low in China, with generally 35% among MDR/RR-TB, which is significantly lower than that in the WHO European Region (84%) (2). It should also be included in resistance screening among rifampicin-sensitive patients, either by phenotypic methods or molecular tools.

Nevertheless, discordance between phenotypic resistance profiles and molecular results has often been reported (38, 39). The reasons may include unfixed mutations missed by phenotypic testing, uncertain breakpoint values in phenotypic testing, and missing mutations in the reference databases (9, 40). By analyzing Geno^R^Pheno^S^ samples, we found that an average of 48.6% of the samples carried unfixed mutations, which may partly explain this inconsistency. A small number of samples were identified as Geno^S^Pheno^R^, which might be caused by rare mutations that are not included in the mutation database. Overall, the concordance between phenotypic testing and genomic prediction of drug resistance in this study is relatively high and satisfactory, similar to the results of large-scale global assessments and meta-analyses (41–43). Collectively, genomic testing for targeted genes can provide timely and sensitive results for population screening or individual monitoring, and the combination of phenotypic and genotypic resistance will more accurately determine the complete resistance profile.

Our study has some limitations. First, this study analyzed routine surveillance data, including resistance screening, in which the collection of clinical samples was limited by site capacity and did not follow a strict research framework. Positive cultures that successfully underwent phenotypic resistance testing accounted for about half of the bacteria-positive patients, which may have biased some estimates. Then, refractory patients were defined as those with two consecutive positive cultures, which were also affected by routine sample collection and laboratory testing. Future work is needed to improve the quality of real-world surveillance systems and ensure the comprehensiveness of clinical samples. In addition, we sequenced strains after the COVID-19 pandemic and could only provide genomic evidence for samples from the past 2 years, making it difficult to confirm the strain identity in earlier times. The number of transmission clusters may also have been underestimated. Besides, clinical data in our study lacked comprehensive details on patients’ lifestyle, nutritional status, and other relevant characteristics, making it difficult to perform a more thorough analysis of potential risk factors.

In summary, this study characterized the molecular features of drug-resistant TB in Xianning, China, and emphasized the severity of resistance accumulation in both initial drug-sensitive and -resistant patients. It is urgent to promote the decentralization of drug-resistant tuberculosis treatment management to reduce the risk of drug resistance accumulation in patients in rural areas with limited medical conditions. By elucidating the genetic and spatial dimensions of TB transmission and drug resistance, this study provides critical insights for shaping evidence-based strategies to combat this persistent global health challenge.

## Data Availability

Data collected for the study include individual participant data and a data dictionary defining each field in the set. These will be made available in the form of deidentified data upon a reasonable request made to the corresponding author. The raw sequencing data of drug-resistant *M. tuberculosis* were deposited on the China National Center for Bioinformation (https://ngdc.cncb.ac.cn/) with the BioProject number of PRJCA033226.
